# Estimation des valeurs normales de l’hémogramme à Marrakech: étude préliminaire au CHU Med VI de Marrakech

**DOI:** 10.11604/pamj.2018.30.249.14648

**Published:** 2018-08-06

**Authors:** Dounya Bounid, Khalil Haouach

**Affiliations:** 1 Laboratoire d’Hématologie Biologique, Hôpital Errazi, CHU Med VI, Marrakech, Faculté de Médecine et de Pharmacie de Marrakech, Maroc

**Keywords:** Valeurs de référence, hémogramme, Marrakech, Reference values, blood cell count, Marrakech

## Abstract

**Introduction:**

L'hémogramme est un des examens les plus prescrits et les plus utiles en pratiques médicales courantes. Ses modifications peuvent révéler des changements très diverses, une interprétation correcte est alors essentielle pour orienter le diagnostic. Le But de ce travail est de déterminer les valeurs de référence de l'hémogramme dans une population d'adultes sains de la ville de Marrakech.

**Méthodes:**

Nous avons effectué une étude portant sur l'analyse de 500 hémogrammes de donneurs adultes reçus au centre de transfusion IBN TOFAIL à Marrakech. Les prélèvements sont analysés par l'automate XE 5000 et les résultats sont analysés par le logiciel statistique SPSS.

**Résultats:**

Les valeurs de référence obtenues: les GB entre 3,9.103/ μl - 10,23.103/ μl (femmes) et 5,11.103/ μl -9,99.103/ μl (hommes), les PNN entre 1,53.103/ μl - 6,05.103/ μl (femmes) et 1,56.103/ μl - 5,97.103/ μl (hommes) et les LYM 1,28.103/ μl - 3,72.103/ μl (femmes) et 1,17.103/ μl - 3,69.103/ μl (hommes). L'HB entre 12,94-14,7g/dl (femmes) et 11,77-18,17g/dl (hommes), les PLQ entre 132.103/ μl - 384.103/ μl (femmes) et 140.103/ μl -356.103/ μl (hommes).

**Conclusion:**

Les données établies au cours de cette étude, fournissent des valeurs de référence préliminaires spécifiques à notre population d'étude, qui pourraient être utilisées pour guider la gestion des patients et l'interprétation des résultats de la recherche clinique et qui peuvent potentiellement améliorer la qualité des soins cliniques offerts aux patients.

## Abstract (en)

**Introduction:**

The full blood count (FBC) is one of the most commonly requested and useful test in common medical practices. Abnormalities can indicate the presence of many disorders. A correct interpretation is then essential to guide the diagnosis. This study aims to determine the reference ranges for full blood count (FBC) in a population of healthy adults in the city of Marrakech.

**Methods:**

We conducted a study analysis of 500 full blood count (FBC) data from adult donors at the Center for blood transfusion Ibn Tofail in Marrakech. The samples were analyzed using XE-5000 automated hematology system and the results were analyzed using SPSS statistical software.

**Results:**

Reference values obtained: white blood cells 3,9.103/ μl - 10,23.103/ μl (women) and 5,11.103/ μl -9,99.103/ μl (men); neutrophils 1,53.103/ μl - 6,05.103/ μl (women) and 1,56.103/ μl - 5,97.103/ μl (men); lymphocytes 1,28.103/ μl - 3,72.103/ μl (women) and 1,17.103/ μl - 3,69.103/ μl (men); hemoglobin 12,94-14,7g/dl (women) and 11,77-18,17g/dl (men); platelets 132.103/ μl - 384.103/ μl (women) and 140.103/ μl -356.103/ μl (men).

**Conclusion:**

Study data provide preliminary reference ranges for full blood count (FBC) in our study population which could be used to guide the treatment of patients and the interpretation of clinical trial results and which could potentially improve the quality of clinical care provided to patients.

## Introduction

Le concept de valeur de référence a été défini pour la première fois en médecine humaine, il y a plus de 30 ans, pour décrire les fluctuations des variables biologiques dans des groupes de sujets parfaitement caractérisés. Ces valeurs de référence sont un outil indispensable à la biologie médicale et notamment en hématologie. Cette notion a ensuite été travaillée et mise à jour pour aboutir à la dernière version des recommandations internationales par l'international Fédération of Clinicat Chemiser and Laboratoire Médicine (IFCC) et le Clinicat Laboratory and Standards Institute (CLSI). L'hémogramme est un des examens les plus prescrits et les plus utiles en pratiques médicales courantes. Ses modifications peuvent révéler des pathologies très diverses, une interprétation correcte est alors essentielle pour orienter le diagnostic. Au Maroc, rares les laboratoires qui disposent de ses propres valeurs de référence d'hémogramme. Il nous a semblé pertinent de réaliser une enquête sur une population d'adultes sains, au niveau de la région de Marrakech -Safi, afin de définir au mieux les normales des différents paramètres de l'hémogramme des patients venant au CHU Marrakech. L'objectif de notre étude a été d'établir les valeurs de référence des paramètres de l'hémogramme chez l'adulte sain au Laboratoire d'Hématologie du CHU de Marrakech, dans le but de fournir des valeurs de référence spécifiques à ce laboratoire.

## Méthodes

**Figure 1:**
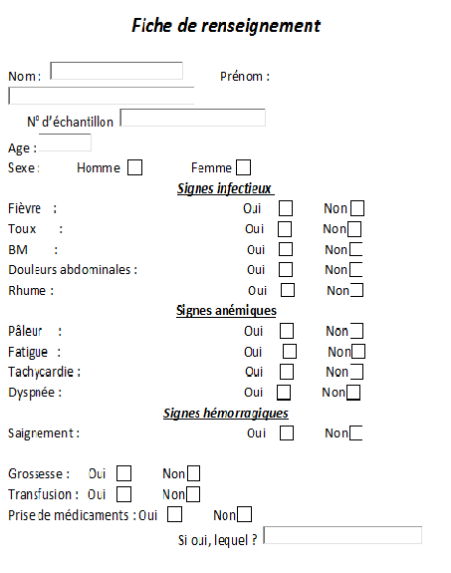
Fiche de renseignement clinique des sujets donneurs de sang

Pour notre enquête, on a fait appel aux donneurs du sang dans le centre de transfusion IBN TOFAIL qui respectent les critères d'une fiche d'exploitation que nous avons établie. Les critères d'inclusion étaient: adultes des 2 sexes, bon état général après examen clinique. Le choix des donneurs repose sur l'éventualité que la population est théoriquement en bonne santé et qu'elle bénéficie d'une consultation médicale avec un interrogatoire pré-don éliminant toute suspicion de maladie. Une fiche de renseignement (Figure 1) propre à chaque donneur est remplie. Elle inclue des renseignements personnels comme le sexe et l'âge, les antécédents de maladie infectieuse virale, bactérienne ou parasitaire, de transfusion, d'intervention chirurgicale, la prise de médicaments… Le consentement a été obtenu pour l'ensemble des participants. Sont inclus dans notre étude, tout donneur de sang; marocain adulte homme et femme; apte au don après la consultation médicale. Les critères d'exclusion étaient: signes infectieux, signes anémiques, signes hémorragiques, grossesse, transfusion et prise de médicaments. Les donneurs sont inscrits dans le registre, le technicien responsable leur attribue un numéro d'ordre identique sur leurs fiches de renseignements, ainsi que sur les tubes. Ce numéro d'ordre permet l'identification du donneur à n'importe quel moment de la chaîne de l'analyse et permet de relier l'échantillon biologique au donneur et au résultat obtenu. Le prélèvement sanguin veineux est réalisé sous vide dans un tube de 4 ml avec comme anticoagulant l'acide éthylène diamine tétra-acétique dipotassique (EDTA). Une fois les prélèvements réalisés, ils sont expédiés dans les plus brefs délais, dans des portoirs au laboratoire d'hématologie et d'immuno-hématologie pour être analysés et ceci dans les règles de sécurité du personnel et d'intégrité de l'échantillon.

La numération sanguine des échantillons s'est réalisée sur l'automate XE-5000i. L'analyseur XE-5000 est un analyseur d'hématologie caractérisé par sa rapidité et son utilité. Il a une capacité forte de détection des échantillons pathologiques. Il permet aussi d'effectuer une formule en 5 populations ainsi qu'une numération des NRB. La technologie d'analyse par fluoro-cytométrie en flux offre une précision encore jamais atteinte. Extrêmement rapide (jusqu'à 150 échantillons par heure), l'analyseur permet de fournir des résultats dans des délais réduits et ainsi d'augmenter la productivité du laboratoire. La validité des mesures est garantie par le passage à chaque série d'analyse de 3 contrôles de la numération formule sanguine, il existe trois niveaux de test: Level 1: niveau normal; Level 2: niveau pathologique bas; Level 3: niveau pathologique haut. Les paramètres étudiés ont été: 1) les paramètres érythrocytaires: numération érythrocytaire, hémoglobine (HGB), hématocrite (HCT), volume globulaire moyen (VGM), concentration corpusculaire moyenne en hémoglobine (CCMH), teneur globulaire moyenne en hémoglobine (TGMH); 2) les paramètres leucocytaires: numération leucocytaire, formule sanguine (polynucléaires neutrophiles, éosinophiles, basophiles; lymphocytes; monocytes); 3) les paramètres plaquettaires: numération plaquettaire. Les résultats obtenus ont été analysés à l'aide du logiciel IBM SPSS statistics 22, qui permet de calculer les différents paramètres en statistique tels que la moyenne, les écarts types, la variance, la normalité.

## Résultats

**Figure 2:**
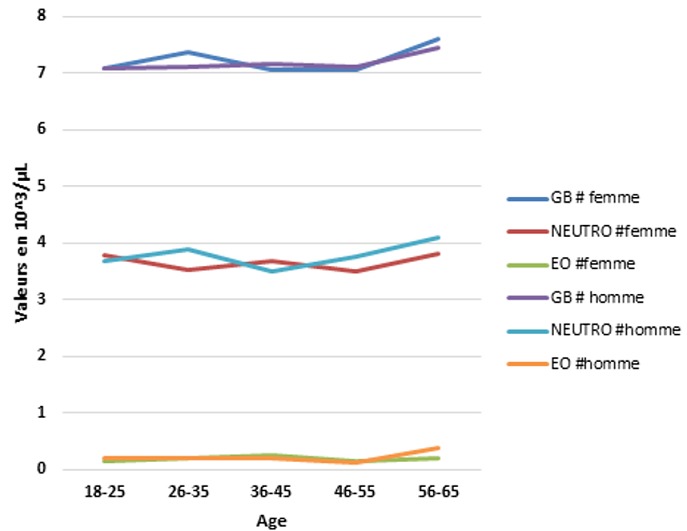
Variation des valeurs moyennes des GB, PNN et PNE en fonction de l’âge et du sexe

**Figure 3:**
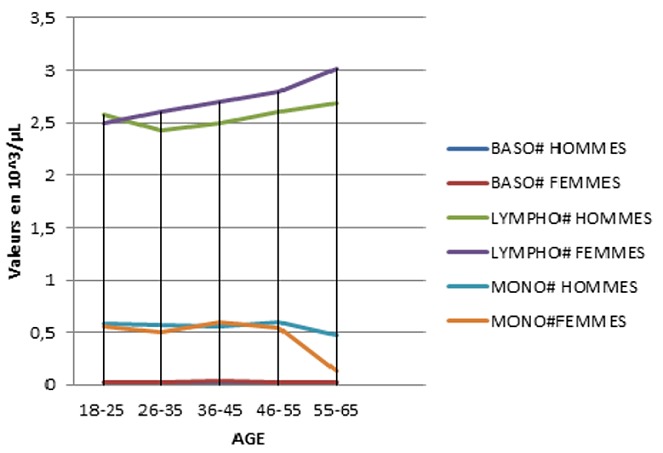
Variation des valeurs moyennes des PNB, lymphocytes et monocytes en fonction de l’âge et du sexe de la population d’étude

**Figure 4:**
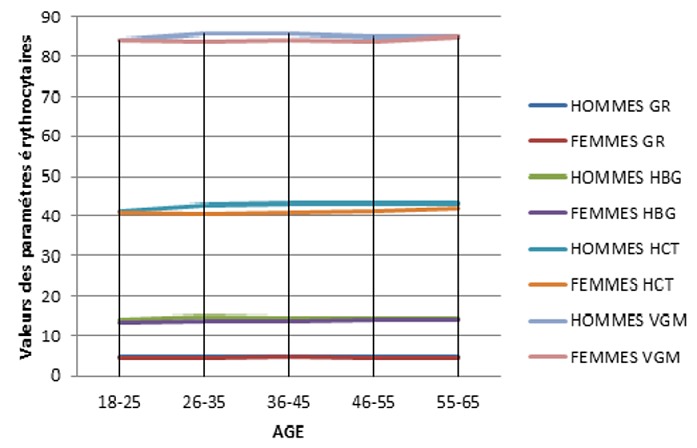
Variation du nombre moyen des globules rouges, le taux d’hémoglobine, l’hématocrite et le VGM en fonction de l’âge et du sexe

**Figure 5:**
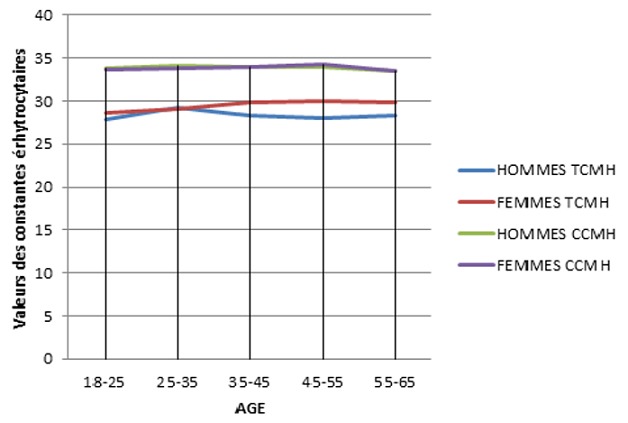
Variation des valeurs moyennes du TCMH et CCMH en fonction de l’âge et du sexe

Parmi les 500 tests effectués, nous n'avons éliminé que 10 (2%), après application des critères d'exclusion. Les tests réalisés concernent 490 prélèvements dont 42,2% effectués sur des femmes et 57,8% sur des hommes (Tableau 1). Nous avons classé les 490 sujets étudiés par tranches d'âge, et nous avons mentionné le résultat de chaque paramètre par sa valeur moyenne encadrée par les deux écart-types: cette étude a été faite chez les candidats de sexe masculin (Tableau 2) et les candidats de sexe féminin (Tableau 3). Des différences significatives selon le sexe et l'âge ont été observées pour tous les paramètres: pour les paramètres leucocytaires: les Figure 2 et Figure 3 reprennent les valeurs des paramètres leucocytaires chez les deux sexes. Le nombre des GB ne montre aucune différence significative entre les deux sexes. Une augmentation discrète est remarquée à partir de l'âge de 55 ans pour les deux sexes. Le nombre absolu des neutrophiles est supérieur chez la femme que l'homme et devient inférieur à l'âge de 35 ans. Il n'a pas été signalé de différence selon le sexe pour les valeurs des numérations des basophiles et des éosinophiles. Le nombre absolu des lymphocytes chez les femmes est inférieur à celui trouvé chez les hommes. A l'âge de 25 ans, il connaît une augmentation pour les deux sexes. Les valeurs du nombre absolu des monocytes ne montrent aucune différence significative entre les deux sexes, les valeurs diminuent discrètement à partir de l'âge de 55 ans pour les deux sexes. Pour les paramètres de la lignée érythrocytaire (Figure 4 et Figure 5).

**Tableau 1:** Répartition de la population étudiée selon les deux sexes

	Fréquence	Pourcentage %
**F**	207	42,2
**H**	283	57,8
**Total**	490	100,0

Les tests réalisés concernent 490 prélèvements dont 42,2 % effectués sur des femmes et 57,8% sur des hommes

**Tableau 2:** Valeurs moyennes d’hémogramme chez les hommes selon l’âge

HOMMES					
AGE (Ans)	18 - 25	26 - 35	36 – 45	46 – 55	56 - 65
GR (10^6/μl)	4.92 ± 0.5	4.96 ± 0.75	4.93 ± 0.63	4.95 ± 0.5	4.92 ± 0.65
HGB (g/dl)	14.03 ± 1.2	14.7 ± 1	14.3 ± 1.1	14.5 ± 1.5	14.42 ± 1.2
HCT (%)	41.24 ± 3.8	42.86 ± 3.4	43.01 ± 2.8	43.2 ± 3.4	43.23 ± 3
VGM (Fl)	84.01 ± 4.9	85.61 ± 4.8	85.5 ± 4.5	85.08 ± 4.6	84.71 ± 4.6
TCMH (pg)	27.83 ± 2.8	29.2 ± 2.7	28.3 ± 2.5	28 ± 2.6	28.36 ± 2.7
CCMH (g/dl)	33.75 ± 1.7	34.03 ± 1.5	33.97 ± 2	34 ± 0.99	33.43 ± 1.2
PLQ (10^3/ Ul)	250 ± 54	249 ± 55	250 ± 50	245 ± 48	240 ± 51
GB (10^3/ Ul)	7.08 ± 0.65	7.12 ± 0.55	7.15 ± 0.75	7.1 ± 0.56	7.44 ± 0.5
NEUTRO# (10^3/ Ul)	3.68 ± 0.99	3.88 ± 1	3.5 ± 0.85	3.76 ± 1.05	4.1 ± 1
LYMPHO# (10^3/ Ul)	2.57 ± 0.75	2.42 ± 0.5	2.5 ± 0.6	2.6 ± 0.9	2.68 ± 0.5
MONO# (10^3/ Ul)	0.59 ± 0.23	0.57 ± 0.2	0.56 ± 0.2	0.6 ± 0.32	0.47 ± 0.2
EO# (10^3/ Ul)	0.2 ± 0.1	0.19 ± 0.1	0.2 ± 0.15	0.11 ± 0.16	0.37 ± 0.14
BASO# (10^3/ Ul)	0.025 ± 0.01	0.03 ± 0.05	0.03 ± 0.07	0.03 ± 0.05	0.03 ± 0.04

Une augmentation discrète des valeurs est remarquée à partir de l’âge de 55 ans pour les hommes.

A l’âge de 25 ans le nombre absolu des lymphocytes connaît une augmentation pour les hommes.

Les valeurs du nombre absolu des monocytes diminuent discrètement à partir de l’âge de 55 ans pour les hommes

**Tableau 3:** Valeurs moyennes d’hémogramme chez les femmes selon l’âge

FEMMES					
AGE (ans)	18 - 25	26 - 35	36 – 45	46 - 55	56 - 65
GR (10^6/Ul)	4.52 ± 0.3	4.41 ±0.5	4.62 ± 0.4	4.55 ± 0.3	4.53 ± 0.3
HGB (g/dl)	13.5 ± 0.94	13.58 ± 1.3	13.88 ± 0.99.	13.96 ±1.5	13.97 ± 1.2
HCT (%)	40.88 ± 2.79	41.08 ± 1.99	40.9 ± 2.6	41.2 ±2.4	41.05 ±1.85
VGM (fL)	84 ± 3.99	83.9 ±4.01	84.1 ± 3.1	83.88 ± 2.99	84.3 ±2.56
TCMH (pg)	28.57 ± 1.94	29.2 ± 1.5	29.8 ± 2.01	29.99 ± 1.6	29.8 ± 1.75
CCMH (g/dl)	33.64 ±0.86	33.8 ± 0.76	34.01 ± 1.02	34.3 ± 0.5	33.5 ± 0.96
PLQ (10^3/ Ul)	250 ± 53	255 ± 56	255 ± 46	255 ± 49	249 ± 52
GB (10^3/ Ul)	7.08 ± 0.88	7.36 ±0.52	7.07 ± 0.6	7.05 ± 0.76	7.6 ± 0.7
NEUTRO# (10^3/ Ul)	3.79 ± 1.2	3.52 ± 0.98	3.69 ± 0.76	3.50 ± 1.13	3.8 ± 0.86
LYMPHO# (10^3/ Ul)	2.5 ± 0.77	2.6 ± 0.65	2.7 ± 0.98	2.8 ± 0.62	3.01 ± 0.54
MONO# (10^3/ Ul)	0.56 ± 0.15	0.5 ±0.10	0.6 ± 0.13	0.55 ± 0.2	0.65 ± 0.14
EO# (10^3/ Ul)	0.15 ±0.11	0.2 ± 0.03	0.25 ± 0.075	0.14 ± 0.1	0.19 ± 0.05
BASO# (10^3/ Ul)	0.03 ±0.02	0.03± 0.01	0.035 ± 0.015	0.025 ± 0.01	0.03 ± 0.01

Une augmentation discrète des valeurs est remarquée à partir de l’âge de 55 ans pour les hommes.

A l’âge de 25 ans le nombre absolu des lymphocytes connaît une augmentation pour les hommes.

Les valeurs du nombre absolu des monocytes diminuent discrètement à partir de l’âge de 55 ans pour les hommes

**Figure 6:**
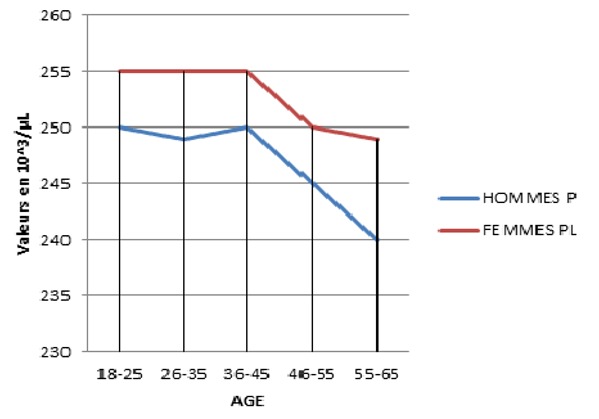
Variation des valeurs moyennes du nombre de plaquettes en fonction de l’âge et du sexe

Le nombre des GR et le taux d'hémoglobine et d'hématocrite sont élevés chez l'homme que chez la femme quel que soit la tranche d'âge. Chez la femme, le taux d'hémoglobines augmente avec l'âge. Le taux d'hématocrite augmente avec l'âge chez l'homme, et une augmentation discrète est signalée chez la femme à l'âge de 45 ans. Le VGM est supérieur chez l'homme que chez la femme. Le TCMH est inférieur chez l'homme par rapport à la femme mais à partir de l'âge de 35 ans, la valeur moyenne du TCMH est la même pour les deux sexes. Le CCMH ne connaît pas de différence significative en fonction de l'âge et du sexe. Pour les valeurs des plaquettes (Figure 6), le nombre des plaquettes évolue simultanément pour les deux sexes, il est inférieur chez la femme que l'homme jusqu'à l'âge de 35 ans. Une diminution discrète est signalée à l'âge de 45 ans pour les deux sexes. Les moyennes des globules rouges, de l'hémoglobine, de l'hématocrite et des constantes érythrocytaires ont été significativement plus élevées chez les hommes par rapport aux femmes. Le nombre de ces paramètres a une légère tendance à augmenter en fonction de l'âge chez les deux sexes. La numération leucocytaire a été significativement plus importante chez les femmes, avec une diminution significative en fonction de l'âge. Les femmes ont des valeurs de polynucléaires neutrophiles qui sont significativement plus élevées que les hommes et aucune différence n'est signalée pour les valeurs des éosinophiles et les basophiles chez les deux sexes. Par contre, les taux de monocytes deviennent significativement plus bas chez la femme par rapport à l'homme à partir de l'âge de 55 ans. On a noté aussi une augmentation significative de la lymphocytose avec l'âge dans les deux sexes. La numération plaquettaire quant à elle a été significativement plus élevée chez les femmes.

## Discussion

Les organismes internationaux comme l'International Council For Standardization in Haematology (ICSH), l'Organisation Mondiale de la Santé (OMS), le Clinical and Laboratory Standards Institute (CLSI) et l'IFCCS-LM définissent la VR comme étant la valeur obtenue par l'observation ou la mesure d'une quantité définie sur un individu de référence. L'ensemble de ces valeurs pour une même quantité constitue l'intervalle de référence. Ce dernier est défini par une valeur supérieure et une autre inférieure qui ne sont autres que les limites de référence, mais dans certains cas, seule la limite de référence inférieure ou supérieure peut être retenue [1]. Les études menées dans différents pays convergent toutes sur la variabilité des VR des paramètres hématologiques et biochimiques selon plusieurs facteurs comme l'âge, le sexe, l'origine ethnique, la grossesse, le tabagisme ou la consommation d'alcool ou de médicament [2, 3]. Pour cela, les recommandations des organismes soulignent l'intérêt de la détermination des VR pour chaque sous type (femme/homme ou enfant/adulte) mais également pour chaque région voire chaque laboratoire parce qu'il faut considérer la race comme facteur pouvant subdiviser un intervalle [4]. Les VR sont déterminées sur des prélèvements réalisés chez un individu de référence, c'est-à-dire une personne sélectionnée selon des critères bien définis et qui sera la base de l'échantillon de référence [1, 5]. Vu la disparité des valeurs de référence disponibles dans la littérature, on a été contraint de comparer nos résultats, d'une part aux valeurs de référence utilisées dans le Laboratoire d'Hématologie du CHU Marrakech (CHUM) (Tableau 4) et d'autre part aux résultats de plusieurs études effectués à l'échelle national et international (Tableau 5) et Tableau 5 (suite) [6-11].

**Tableau 4:** Intervalles de référence de notre étude comparés aux intervalles de référence du laboratoire de CHUM

	Valeurs utilisées au CHUM		Valeurs de notre étude	
	HOMMES	FEMMES	HOMMES	FEMMES
GR (10^6/μl)	4.4 -5.5	4 - 5.50	4.2 – 5,98	3,94 – 5,7
HGB (g/dl)	14 - 16	11.5 - 15	11,77 - 18,17	12,94 – 14,7
HCT (%)	40 – 50	38 - 45	34,91-53,07	31,88 - 49,8
VGM (fL)	85 – 95	85 - 95	73,08 – 96	71,4 – 96,6
TCMH (pg)	27 à 32	27 à 32	24-33,6	24,80 - 32,30
CCMH (g/dl)	32 - 36	32 - 36	31,8- 36,16	31,2 – 36,06
PLQ (10^3/ μl )	150 - 400	150 - 400	140 – 356	132 – 384
GB (10^3/ μl)	4 – 10	04-10	5,11 - 9 ,99	3,9 – 10,23
NEUTRO# (10^3/ μl)	02-07	02-07	1,56 - 5,97	1,53 – 6,05
LYMPHO# (10^3/ μl)	0.9 - 5.2	0.9 - 5.2	1,17 - 3,69	1,28 – 3,72
MONO# (10^3/ μl)	0.1 - 1	0.1 - 1	0,22 - 0,9	0,14 – 0,98
EO# (10^3/ μl)	0.05 - 0.3	0.05 - 0.3	0,06 - 0,38	0,13 – 0,43
BASO# (10^3/ μl)	0.01 - 0.05	0.01 - 0.05	0,06 - 0,38	0,01 – 0,07

Les intervalles de référence de notre étude sont plus larges que ceux utilisés par le Laboratoire du CHUM. Notre étude a permis d’établir des valeurs minimales inférieures à celles du Laboratoire pour les GR, HGB, HCT, VGM, TCMH, CCMH, PLQ.

D’autre part, les intervalles de référence du laboratoire sont plus larges que les nôtres, pour les paramètres leucocytaires: nos valeurs minimales sont supérieures à celles du laboratoire et nos valeurs maximales sont inférieures à celles du Laboratoire

**Tableau 5:** Intervalles de référence de notre étude comparés aux intervalles de référence d’autres études

	NOTRE ETUDE		France		GHANA	
SEXE	FEMME	HOMME	FEMME	HOMME	FEMME	HOMME
GR (10^6/Ul)	3,94-5,7	4,2-5,98	3,96-5,12	4,39-5,68	3,09-5,30	3,79-5,96
HGB (g/dl)	12,94-14,7	11,77-18,17	11,70-15,00	13,40-16,70	8,80-14,40	11,30-16,40
HCT (%)	31,88-49,8	34,91-53,07	34,70-44,40	39,20-48,60	26,40-45,00	33,20-50,50
VGM (fL)	71,4-96,6	73,08-96	78,40-95,30	80,20-95	73,00-96,00	70-98
TCMH (pg)	24,80-32,30	24-33,6	26,10-32,50	27,20-32,80	22,30-33,60	22,70-33,50
CCMH (g/dl)	31,2-36,06	31,8-36,16	31,90-35,80	32,40-36,30	30,40-36,50	30,60-36
PLQ (10^3/ Ul)	132-3840	140-356	186-440	171-397	89-403	88-352
GB (10^3/ Ul)	3,9-10,23	5,11-9 ,99	3,91-10,88	4,08-10,81	3,40-9,30	3,50-9,20
NEUTRO# (10^3/Ul	1,53-6,05	1,56-5,97	1,74-7,10	1,82-6,81	1,50-5,60	1,50-5,90
LYMPHO# (10^3/Ul	1,28-3,72	1,17-3,69	1,26-3,64	1,27-3,77	1,20-4,40	1,20-5,20
MONO# (10^3/ Ul)	0,14-0,98	0,22-0,9	0,20-0,66	0,23-0,74	0,20-0,90	0,20-1,40
EO# (10^3/ Ul)	0,13-0,43	0,06-0,38	0,04-0,52	0,04-0,56	-	-
BASO# (10^3/ Ul)	0,01-0,07	0,01-0.07	0-0,08	0-0,09	-	-

**Tableau 5 (suite):** Intervalles de référence de notre étude comparés aux intervalles de référence d’autres études

BINET		RABAT		MADAGASCAR		MALYSIA	
FEMME	HOMME	FEMME	HOMME	HOMME	FEMME	FEMME	HOMME
-	-	3,9-5,96	4,83-7,03	5,3+/-0,01	4,7+/-0,01	4,34(0,41)	5,12(0,47)
12-16	13-18	10,10-16,97	13,8-20,21	156+/-15,4	135,8+/-20,9	11,83(1,01)	1,27(1,13)
-	-	31,39-51,64	43,38-61,60	46,6+/-4,9	40,6+/-6,7	37,08(2,66)	43,62(3,07)
82-98	82-98	74,00-100,08	77,97-100,56	87,4+/-11,4	86,4+/-11,6	85,99(4,25)	87,32(4,21)
27-32	27-32	19,91-31,97	24,80-32,30	337,8+/-19,6	334,7+/-2,1	31,90(1,23)	32,70(1,06)
32-36	32-36	27,50-33,90	29,68-34,20	29,7+/-4,3	29,03+/-4,5	27,99(1,62)	28,24(1,43)
150-400	150-400	121,13-341	108-327,25	249+/-40	275+/-31	275,24(63,47)	254,9(43,67)
4-10	4-10	5,10-11,58	4,60-13,81	5,23+/- 2,74	6,28+/-3,22	6,73(1,68)	6,74(1,48)
1,5-7	1,5-7	2,05-9,46	1,60-9,80	2,6+/-0,06	3,70+/-0,10	3,81(1,13)	3,76(1,09)
1,5-4	1,5-4	0,42-4,18	0,59-4,41	1,96+/-0,03	1,96+/-0,03	2,17(0,56)	2,18(0,52)
0,1-1	0,1-1	0-1,18	0-1,44	0,36+/-0,03	039+/-0,0	0,42(0,16)	0,41(0,13)
0,05-0,5	0,05-0,5	0-0,80	0-0,80	0,23/-004	0,18+/-0,03	0,15(0,12)	0,18(0,10)
0,01-0,05	0,01-0,05	0-0,20	0-0,50	0,023+/-0,001	0,031+/-0,006	0,03(0,02)	0,03(0,02)

Nos résultats pour la majorité des paramètres hématologiques sont différents des autres données publiées, ce qui prouve qu'un développement local des gammes de référence est essentiel pour tout laboratoire

Les intervalles de référence de notre étude sont plus larges que ceux utilisés par le Laboratoire du CHUM. Notre étude a permis d'établir des valeurs minimales inférieures à celles du Laboratoire pour les GR, HGB, HCT, VGM, TCMH, CCMH, PLQ. D'autre part, les intervalles de référence du laboratoire sont plus larges que les nôtres, pour les paramètres leucocytaires: nos valeurs minimales sont supérieures à celles du laboratoire et nos valeurs maximales sont inférieures à celles du Laboratoire. Les résultats de notre étude ont montré qu'il existait des différences significatives dans les valeurs de la lignée érythrocytaire, entre la population masculine et féminine, chose qui est très souvent rapportée dans la littérature [12, 13] et qui est expliquée par des pertes physiologiques en fer plus importantes chez la femme d'une part, et par une stimulation érythropoiétique d'origine androgénique plus importante chez l'homme d'autre part [14]. Nos résultats pour la majorité des paramètres hématologiques sont différents des autres données publiées, ce qui prouve qu´un développement local des gammes de référence est essentiel pour tout laboratoire. Notre échantillonnage direct a concerné 500 donneurs qui se sont présentés au centre de transfusion de l'hôpital IBN TOFAIL Marrakech. Notre effectif est validé selon les recommandations internationales de l'IFCC-LM et le CLSI (nombre d'individus ≥ 120) concernant le nombre. Mais ces recommandations précisent que ce nombre est exigé pour chaque groupe [1]. Sachant que notre échantillonnage est sélectif, on pense qu'il serait judicieux de choisir d'autres groupes de la population marocaine pour relever les différences et les similitudes dans les différentes catégories de notre population.

Marrakech, situé au centre du Maroc, bénéficie d'un climat semi-aride, la population marrakchie vit à une altitude à 457m. Même si certaines variations spécifiques, notamment liées aux différentes caractéristiques ethniques, ne peuvent être ignorées, on peut considérer que les individus vivant à Marrakech ne présentent aucune variation imputable à leur lieu de vie ou au lieu de prélèvement de leur échantillon sanguin. Nous avons présenté les données normales de l'hémogramme obtenues entre avril et juillet 2017 sur une population vivant à Marrakech, avec des conditions pré analytiques établies et des méthodes analytiques modernes. Pour des raisons méthodologiques, cette étude couvre uniquement les adultes et n'inclut pas d'enfants. Le questionnaire utilisé en pré-prélèvement ne tient pas compte de l'exposition aux facteurs environnementaux, radiations, ni des facteurs génétiques susceptibles de modifier certains facteurs hématologiques. Notre étude est également limitée par l'impossibilité d'éliminer de notre population les sujets présentant une carence en fer ou atteints d'hémoglobinopathies. Notre étude n'a pas pris en considération les valeurs des réticulocytes vu que ce n'est pas un examen systématique, hors cas d'anémie suspectée. Les valeurs fournies par les automates et dépendant des techniques analytiques ou du mode d'expression des résultats défini par le fabriquant, notamment l'indice de répartition des hématies et les valeurs plaquettaires n'ont pas été analysées.

## Conclusion

L'hémogramme est un des examens biologique les plus prescrits et parmi les plus utiles en pratique médicale courante. Ses modifications peuvent révéler des pathologies très diverses. Ses valeurs de référence se voient changer en fonction de plusieurs paramètres comme l'âge, le sexe, le tabagisme mais aussi l'origine ethnique, la grossesse et la consommation de médicaments ou d'alcool. La combinaison fréquente de ces facteurs incitent à réaliser des valeurs de référence pour chaque population voire pour chaque laboratoire d'analyse pour une meilleure interprétation des résultats. Notre étude réalisée au niveau du laboratoire d'hématologie et d'immuno-hématologie en collaboration avec le centre de transfusion sanguine au CHU Med VI de Marrakech a permis de déceler des différences par rapport aux valeurs de référence couramment utilisées.

### Etat des connaissances actuelles sur le sujet

- L'hémogramme est l'examen biologique le plus prescrit et oriente vers des diagnostics multiples;
- Les valeurs de référence varient d'une population à l'autre vu l'origine ethnique, la génétique, la variation environnementale, la nourriture, le tabagisme;
- La non connaissance des valeurs locales conduit à des interprétations erronées et peut inquiéter inutilement les patients.

### Contribution de notre étude à la connaissance

- Une meilleure interprétation des hémogrammes de nos patients puisque les seuils des valeurs de référence sont différents d'une population à une autre et par conséquent une prise en charge adéquate des affections hématologiques et non hématologiques;
- La biologie médicale est actuellement en progression ce qui nécessite une restructuration. Cependant tous les laboratoires sont tenus de faire la preuve de leurs procédures d'accréditation et, par conséquent, de disposer de valeurs de référence reconnues et validées.

## Conflits d’intérêts

Les auteurs déclarent aucun conflit d'intérêts.
